# Quinazolinones, the Winning Horse in Drug Discovery

**DOI:** 10.3390/molecules28030978

**Published:** 2023-01-18

**Authors:** Aishah M. Alsibaee, Hanan M. Al-Yousef, Huda S. Al-Salem

**Affiliations:** 1 Department of Pharmaceutical Chemistry, College of Pharmacy, King Saud University, Riyadh 11564, Saudi Arabia; 2 Department of Pharmacognosy, College of Pharmacy, King Saud University, Riyadh 11564, Saudi Arabia

**Keywords:** 4-(3*H*)-quinazolinones, structure activity relationship, anti-tuberculosis, chemical synthesis, dihydrofolate reductase inhibitor, protein kinase inhibitor

## Abstract

Quinazolines are nitrogen-containing heterocycles that consist of a benzene ring fused with a pyrimidine ring. Quinazolinones, oxidized quinazolines, are promising compounds with a wide range of biological activities. In the pharmaceutical field, quinazolinones are the building blocks of more than 150 naturally occurring alkaloids isolated from different plants, microorganisms, and animals. Scientists give a continuous interest in this moiety due to their stability and relatively easy methods for preparation. Their lipophilicity is another reason for this interest as it helps quinazolinones in penetration through the blood–brain barrier which makes them suitable for targeting different central nervous system diseases. Various modifications to the substitutions around the quinazolinone system changed their biological activity significantly due to changes in their physicochemical properties. Structure–activity relationship (SAR) studies of quinazolinone revealed that positions 2, 6, and 8 of the ring systems are significant for different pharmacological activities. In addition, it has been suggested that the addition of different heterocyclic moieties at position 3 could increase activity. In this review, we will highlight the chemical properties of quinazolinones, including their chemical reactions and different methods for their preparation. Moreover, we will try to modify some of the old SAR studies according to their updated biological activities in the last twelve years.

## 1. Introduction

Quinazolines are fused heterocycles with a wide range of biological activities [1,2]. Nitrogen heterocycles exhibit diverse biological activities due to their similarities with many natural products. Naturally occurring and synthetic quinazolines have been an interesting area of research due to their diverse pharmacological activities; namely, anticancer, immunotropic, hypolipidemic, antiplatelet, hypotensive, antifungal, antimicrobial, and anticonvulsant activities. In the pharmaceutical field, the quinazoline moiety is considered a “privileged structure” for drug development. Moreover, quinazolinones are the building blocks of more than 150 naturally occurring alkaloids isolated from different plants, microorganisms, and animals [3,4]. This review focused on 4(3*H*)-quinazolinones owing to their various approvals for different biological and pharmacological activities. It provides researchers with the most important information about quinazolinones, providing a deeper understanding of such compounds in the field of drug discovery. 

## 2. Chemistry of Quinazolinones

### 2.1. Classification

The name quinazoline (**1**) was proposed in 1887 by Widdege upon the observation that it was isomeric with the compounds cinnoline and quinoxaline. Other names for this heterocycle system are phenmiazine, benzyleneamidine, benzo-1,3- diazine, 5,6-benzopyrimidine, and 1,3-diazanaphthalene. In 1889, Paal and Bush suggested the numbering of the quinazoline ring system [1,5]. Quinazolines are nitrogen-containing heterocycles that consist of a benzene ring fused with a pyrimidine ring. The properties of the pyrimidine ring are considerably altered by the presence of a fused benzene ring. The two nitrogen atoms in the pyrimidine ring are not equivalent, and the polarization of the 3,4-double bond is reflected in the reactions of the quinazoline. Many factors affect the properties of substituted quinazolines, and this depends mainly on the nature of the substituents, the position of the substituents (whether they are on the benzene or the pyrimidine ring), and the conjugation on the pyrimidine ring [5,6].

**Figure 1:**
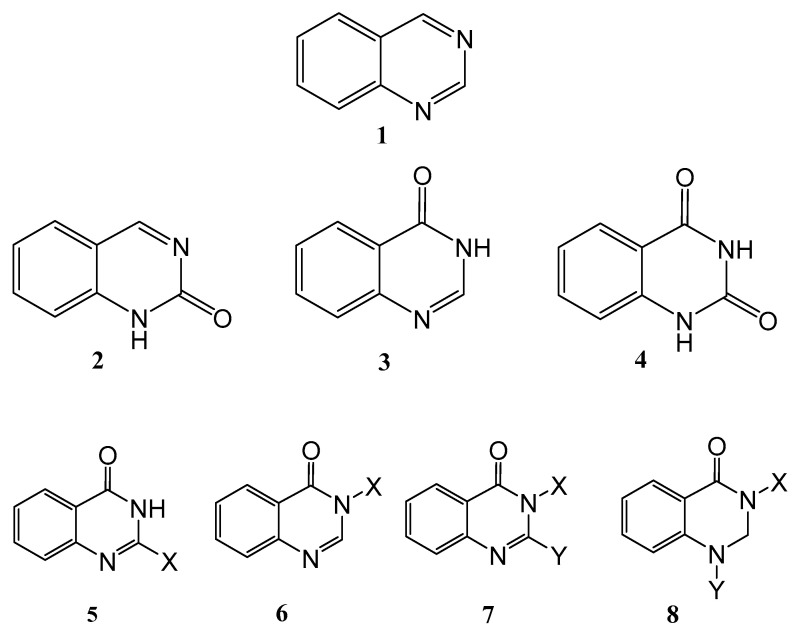
Classification of quinazolines.

On the other hand, quinazolinones are the oxidized form of quinazolines and are the most prominent part of the quinazoline alkaloids. Structure-activity relationship (SAR) studies of quinazolinone revealed that positions 2, 6, and 8 of the ring system are significant due to different pharmacological activities. In addition, it has been suggested that the addition of different heterocyclic moieties at position 3 could increase activity [1]. In Figure 1 quinazolinones are classified according to the position of the oxo group into three types: 2(1*H*(quinazolinone (**2**), 4(3*H*)quinazolinone (**3**), and 2,4(1*H*,3*H*)quinazoline-dione (**4**). Depending on the substitution patterns, they are further classified into 2-substituted-4(3*H*)-quinazolinones (**5**), 3-substituted-4(3*H*)-quinazolinones (**6**), 2,3-disubstituted -4(3*H*)-quinazolinones (**7**), and 2,4-disubstituted-4(3*H*)-quinazolinones (**8**). Substitutions on the benzene ring are also common in many derivatives [6]. 4(3*H*)-quinazolinone (**3**) is one of the most prevalent natural products, and it exists midway in many biosynthetic pathways [3]. Additionally, it has been reported that the quinazolinone ring is quite stable in many reactions such as oxidation, reduction, and hydrolysis [1]. They are stable in cold dilute acid and alkaline solutions, but they are destroyed if the solution is boiled [6]. 

**Scheme 1:**
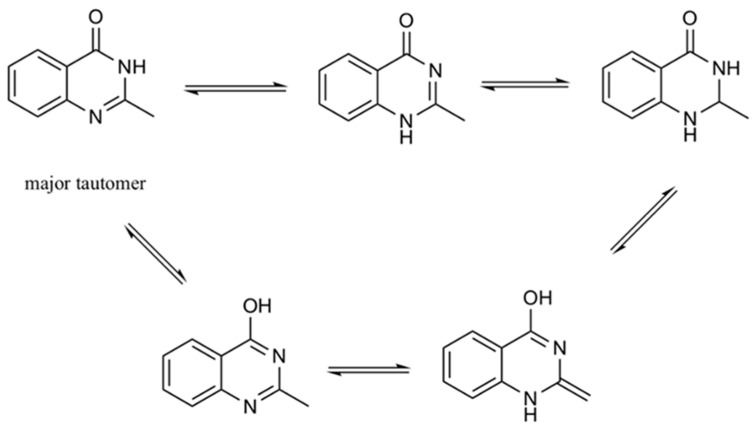
Tautomeric states of 2-methyl-4(3*H*)-quinazolinone [3].

Furthermore, quinazolinones have a strong lactam–lactim tautomeric interaction (Scheme 1). This tautomeric interaction is significant when a 4(3*H*)-quinazolinone containing a methyl in the 3-position is subjected to chlorination with POCl_3_. Chlorination proceeds because the methyl group is lost, but when the methyl group is present in the 2-position, the tautomeric effect is extended by generating an exomethylene carbon. These extended tautomeric effects are significant because they enhance the reactivity of the substituted 4(3*H*)-quinazolinones [3].

Chemical reactions of 4(3*H*)-quinazolinones 

#### 2.1.1. Electrophilic Attack on Carbon (Nitration)

**Scheme 2:**
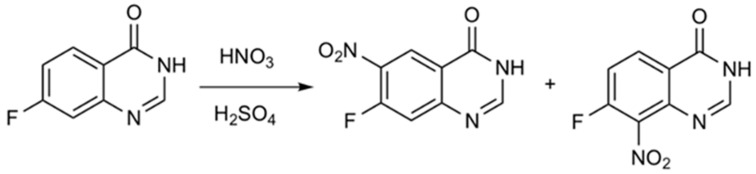
Nitration of 7-fluoro-4(3*H*)-quinazolinone [7].

4(3*H*)-Quinazolinones are nitrated in position 6, but when an ortho-directing substituent is present at the 7-position, an 8-nitrated product is also obtained. Thus 7-fluoro-4(3*H*)-quinazolinone gives a mixture of the 6- and 8-nitro products, although 8-nitro-7-fluoro-4(3*H*)-quinazolinone predominates (Scheme 2) [7]. 

#### 2.1.2. Electrophilic Substitution (Chlorination)

**Scheme 3:**
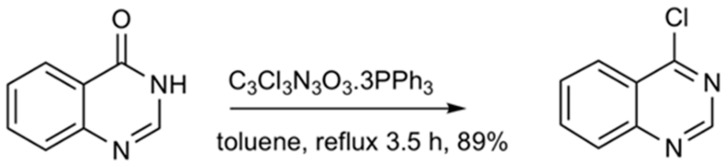
Chlorination of 4(3*H*)-quinazolinone using triphenylphophine and trichloroisocyanuric acid [7].

Hydroxy groups in 4(3*H*)-quinazolinone are substituted by chlorine upon heating with phosphoryl chloride. Thionyl chloride can also be used as the chloride source if used in a catalytic amount of dimethylformamide (DMF). Oxalyl chloride and catalytic (DMF) may also be used. Other reagent systems that have been used include combinations of triphenylphosphine with N-chlorosuccinimide, trichloroisocyanuric acid, and tetrachloromethane (Scheme 3). The reaction of 4(3*H*)- quinazolinone with triphenylphosphine and trichloroisocyanuric acid gave 4-chloroquinazoline in 89% of the yield [7]. 

#### 2.1.3. Reduction of the Pyrimidine Ring

**Scheme 4:**
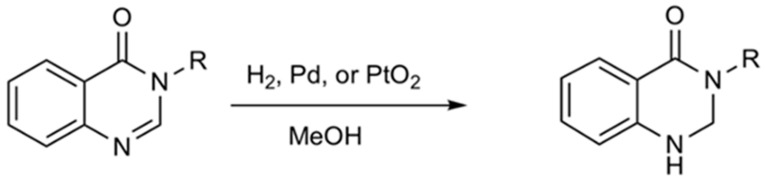
Reduction of the pyrimidine ring of 4(3*H*)-quinazolinone using palladium and platinum oxide [7].

The hydrogenation of 3-substituted 4(3*H*)-quinazolinones has been performed with palladium and platinum oxide to give the 1,2-dihydro derivatives (Scheme 4) [7].

#### 2.1.4. Reduction of the Benzene Ring

**Scheme 5:**
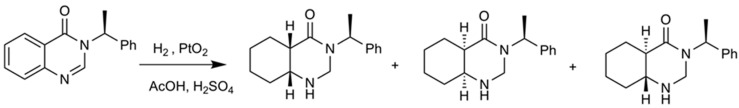
Reduction of the benzene ring of 4(3*H*)-quinazolinone using platinum oxide [7].

The reduction of the benzene ring of 4(3*H*)-quinazolinones can occur with the use of platinum oxide. Scheme 5 shows an example of the reduction of the benzene ring of a chiral 4(3*H*)-quinazolinone. This reaction gives a mixture of the three diastereomeric octahydro-4(1*H*)-quinazolinones [7].

### 2.2. Chemical Synthesis of 4(3H)-Quinazolinones

#### 2.2.1. Griess Synthesis

**Scheme 6:**
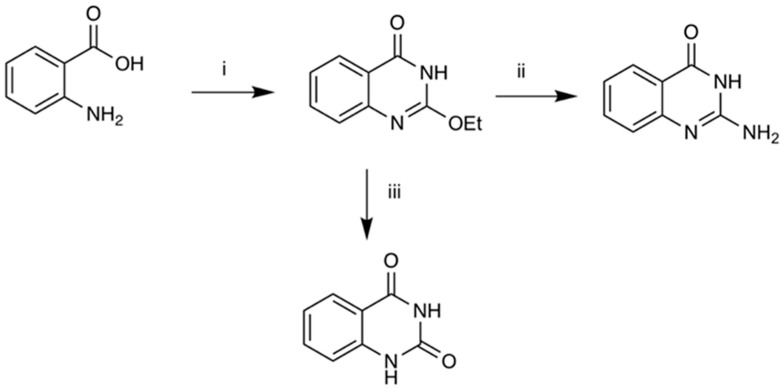
Griess synthesis of 4(3*H*)-quinazolinone and 2-amino-4(3*H*)-quinazolinone and 2,4 (1*H*,3*H*)-quinazilinedione. (i) HCN, EtOH; (ii) NH_4_OH, reflux; (iii) H_2_O [3].

The first reported synthesis of quinazolinone was in 1989 and is known as the Griess synthesis. The first step is the condensation of anthranilic acid and cyanide in ethanol to create 2-ethoxy-4(3*H*)-quinazolinone. This step is followed by a reaction with ammonia to give 2-amino-4(3*H*)-quinazolinone or followed by a reaction with water to give 2,4 (1*H*,3*H*)- quinazilinedione [3] (Scheme 6).

#### 2.2.2. Niementowski Synthesis

**Scheme 7:**
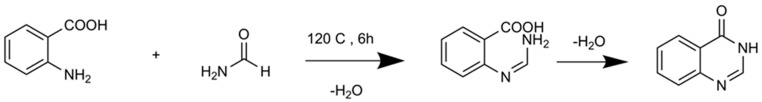
Niementowski synthesis of 4(3*H*)-quinazolinone; Condensation of acetanilides with urethanes [1].

Niementowski synthesis is a simple and easy method to synthesize 4-(3*H*) quinazolinone by the condensation of anthranilic acid with acid amides (Scheme 7) [1,8].

**Scheme 8:**
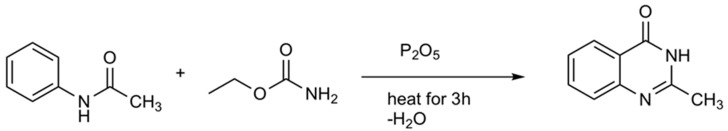
Synthesis of 2-methyl-4(3*H*)-quinazolinone by condensation of acetanilides with urethanes [1].

It is formed by heating anthranilic acid in an open container with excess of formamide at 120 °C. The reaction involves the elimination of water. It proceeds via an *o*-amidobenzamide intermediate. This method has been modified by using microwave irradiation techniques to improve the yield and the reaction time [1,8]. 2-methyl-4-(3*H*(-quinazolinone is synthesized by heating urethane and acetanilide for 3 h with phosphorus pentoxide in toluene (Scheme 8) [1].

#### 2.2.3. Condensation of N-Acylanthranillic Acids with Primary Amines

**Scheme 9:**
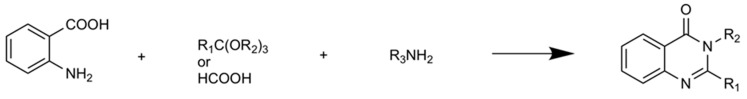
Synthesis of 2,3 disubstituted 4(3*H*)-quinazolinone by condensation of N-acylanthranillic acids with primary amines [1].

This reaction is performed by heating corresponding N-acylanthranillic acid with ammonia or substituted amines. Thereby, much of the primary amines and N-acyl-5-nitroanthranilic acid are condensed to synthesize 2-methyl-3-alkyl-6-nitro-4- (3*H*) quinazolinones in good yields (Scheme 9) [1].

#### 2.2.4. Microwave-Assisted Synthesis Using o-Aminobenzamides

**Scheme 10:**
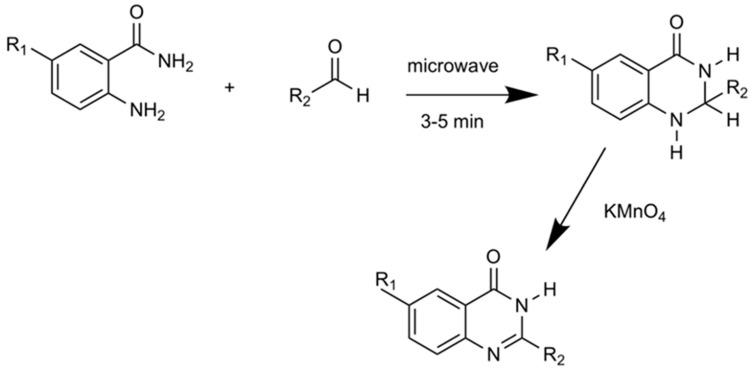
Microwave-assisted synthesis of 4(3*H*)-quinazolinone using o-aminobenzamides [8].

Bakavoli and his group synthesized 4(3*H*(- quinazolinone in one pot using microwave irradiation by the oxidative heterocyclization of *o*-aminobenzamides with aldehydes in the presence of KMnO_4_. This reaction formed a good yield of the targeted 4(3*H*)-quinazolinone (Scheme 10) [8].

## 3. Biological Activities of 4(3*H*)-Quinazolinones

### 3.1. Antimalarial Activity

Febrifugine (**9**), is the active compound extracted from the Chinese medicinal plant *Dichroa febrifuga* Lour. This plant has been prescribed in traditional Chinese medicine for over 2000 years. The antimalarial activity of febrifugine is 100 times more potent than quinine against *Plasmodium lophurae* and 50 times more potent than quinine against *Plasmodium cynomolgi* infection in *rhesus* monkeys. In fact, clinical trials conducted from the 1940s to the 1960s showed that febrifugine caused gastrointestinal irritation and side effects that include diarrhea, vomiting, liver toxicity, and other gastrointestinal tract irritations—major causes for the holdback of this potent antimalarial compound. WHO prevented the approval of this compound or one of its derivatives for the treatment of malaria. Additionally, in the 1960s, different derivatives of febrifugine were developed by the U.S. army including halofuginone (**10**). Further studies of these derivatives were discontinued due to a lack of funding and reliable research approaches [9].

A study by Jiang et al. (2005) discussed the antimalarial activity of febrifugine and its derivatives in vivo. This study investigated the effect of febrifugine and its analogues on different species of parasites that cause malaria in mice. They also explored the effect of different administration routes of the drug for minimizing gastrointestinal side effects. Jiang et al. (2005) concluded that the subcutaneous administration of some of the analogues, such as halofuginone, did not cause gastrointestinal tract irritation [9].

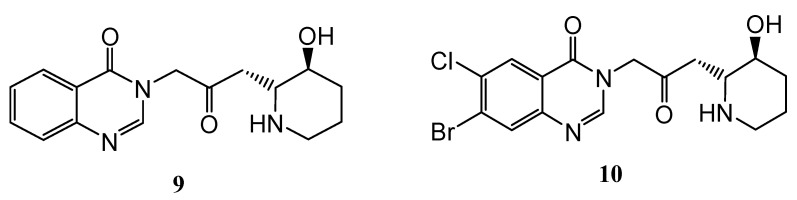



**Scheme 11:**
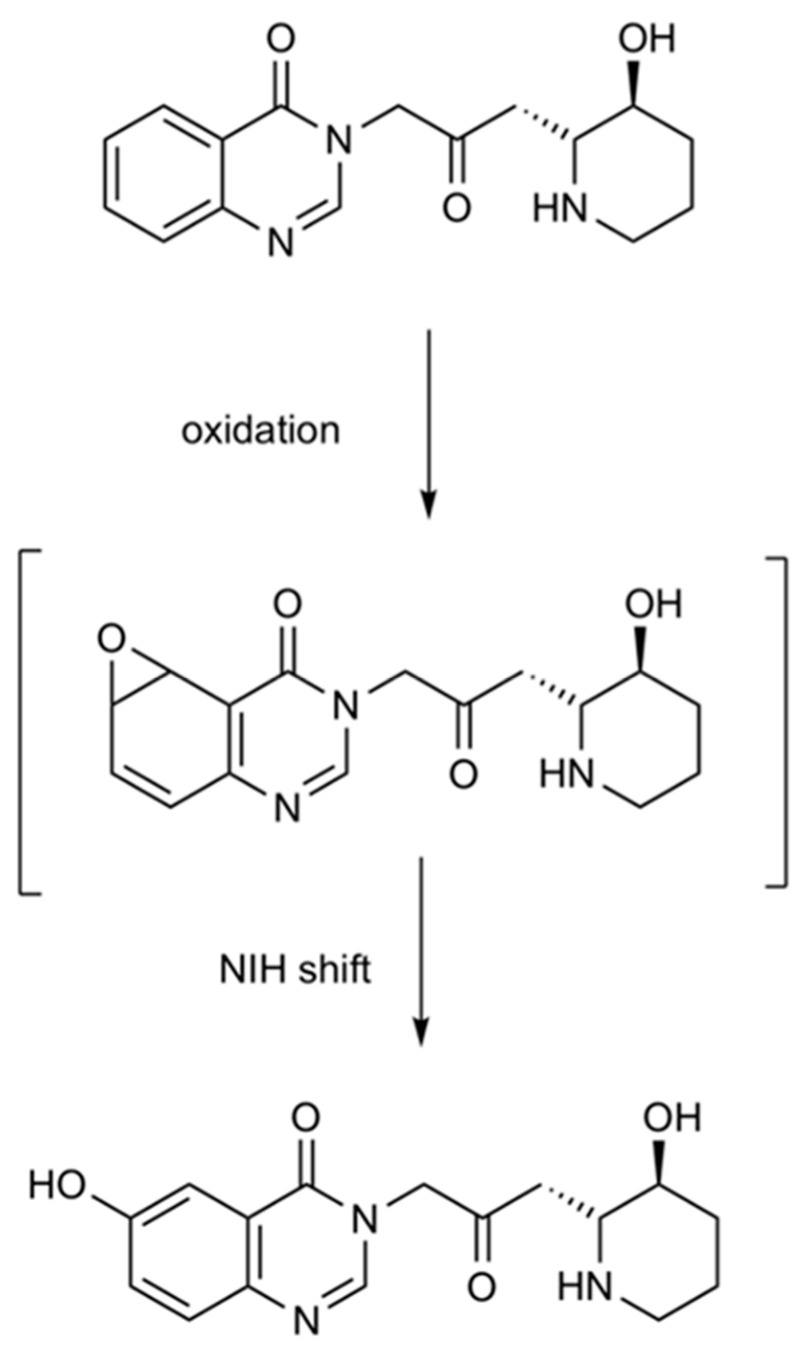
Metabolism of febrifugine to arene oxide metabolite and the NIH shift re-arrangement of arene oxide [10].

Febrifugine could be metabolized by cytochrome P-450 enzymes to the corresponding arene oxide (Scheme 11). This arene oxide can escape the deactivation process by certain enzymes which results in toxicity. The toxicity of this reactive electrophile intermediate is due to its ability to form covalent adducts with the DNA, RNA, and proteins of the host. This binding can cause mutations and result in cell damage. Studies showed that arene oxide intermediate is probably a short-lived reactive metabolic intermediate because it is converted to another metabolite via a rearrangement known as NIH shift [10]. SAR studies have demonstrated that the 4-quinazolinone moiety possesses antimalarial activity, and this is due to both the nitrogen atom of the piperidine ring and the hydroxyl group which are necessary for antimalarial activity [11].

A phenotypic high-throughput screen allowed the design and synthesis of quinazolinone-2-carboxamide derivatives as a new antimalarial lead compound. Structure–activity relationship investigations guided them to prepare a strong inhibitor (**11**), which is 95-fold more potent than the original hit one. Compound **11** has in vitro a fast-killing profile against resistant strains of malaria. The in vivo activity in a murine model of human malaria was very promising too [12].

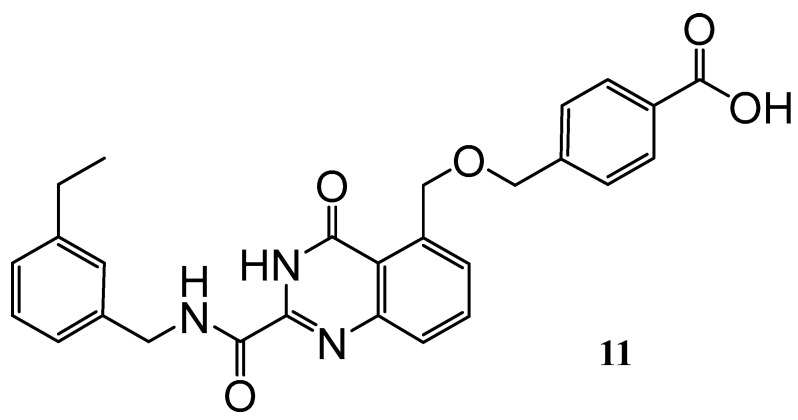



### 3.2. Antituberculosis Activity

Different quinazolinone derivatives have been proven to be effective against *Mycobacterium tuberculosis* bacteria. Isoniazid incorporated 2-styryl-quinazolinone derivatives (**12**), which were synthesized and tested for anti-tuberculosis activity in a study by Babu coworkers [13]. The study tested the compounds’ activity against multi-drug resistant strains of *M. tuberculosis*, and one compound was found to be effective [13].

The authors investigated quinazolinone benzoates as novel anti-tuberculosis agents. The researchers used virtual screening to look for promising anti-tuberculosis agents. They synthesized 24 quinazolinone benzoates and explored their activity. The study found four compounds that displayed significant anti-tuberculosis activity. The authors concluded that quinazolinone benzoates (**13**) are promising candidates against Mycobacterium *tuberculosis* [14].

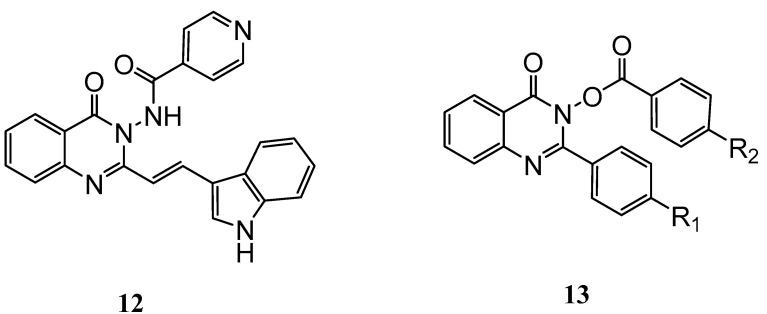



**Figure 2:**
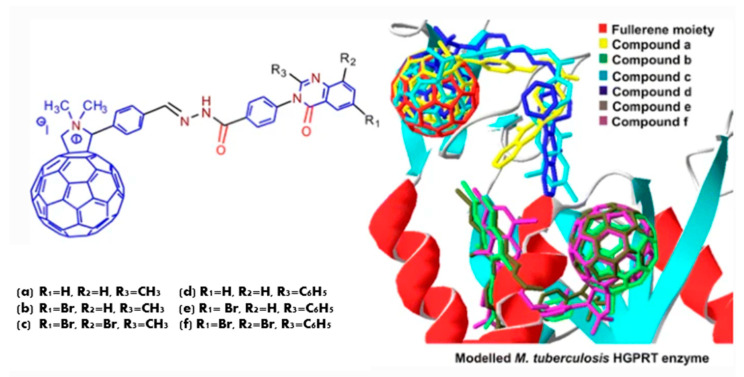
Compound 14 docked into the binding site of *M. tuberculosis* hypoxanthine-guanine phosphoribosyl transferase enzyme [15].

These compounds have different biological activities such as antibacterial and antiviral, as well as possessing cytotoxic effects. A series of cationic fullerene derivatives bearing a substituted quinazolinone moiety were reported as effective antibacterial agents in a 2013 study [15]. The interaction between the compounds and bacterial hypoxanthine-guanine phosphoribosyltransferase was investigated (Figure 2). A combined molecular docking and QSAR study in 2018 was also conducted on similar derivatives of cationic fullerene with quinazolinone moiety [16]. Furthermore, a series of 4(3*H*)-quinazolinones and their fullerene derivatives were computationally investigated. Their binding to 6-oxopurine phosphoribosyl transferase was modeled. The authors (2018) concluded that cationic fullerene derivatives of 4(3*H*)-quinazolinones are significantly more potent against bacteria than quinazolinones. Electrostatic interactions between cationic fullerene derivatives and the enzyme significantly contribute to increasing binding affinity [15,16].

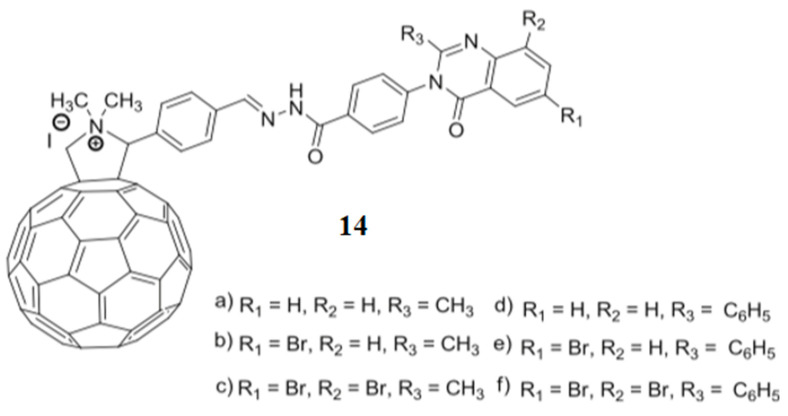



Another 2018 study proved that the effect of the derivatives, such as 2-mercapto-4(3*H)-*quinazolinone scaffold (**15** and **16**), as a potent inhibitor against *mycobacterial* non-proton pumping type II NADH dehydrogenase (NDH-2) in turn plays a critical role in the respiratory metabolism of bacteria. Therefore, SAR studies were performed to understand the activity of potent compounds and have concluded the importance of the quinazolinone core for the activity as well as the S-linker [17]. A more recent 2019 study investigated the anti-tuberculosis activity of 4(3*H*)-quinazolinones linked to 1,2,3-triazole hybrids (**17**). It included the design, synthesis, and docking studies for the proposed agents. It was concluded that the addition of the triazole fragment enhanced the activity of quinazolinone derivatives, whereas the addition of aromatic groups increased the binding interactions [18].

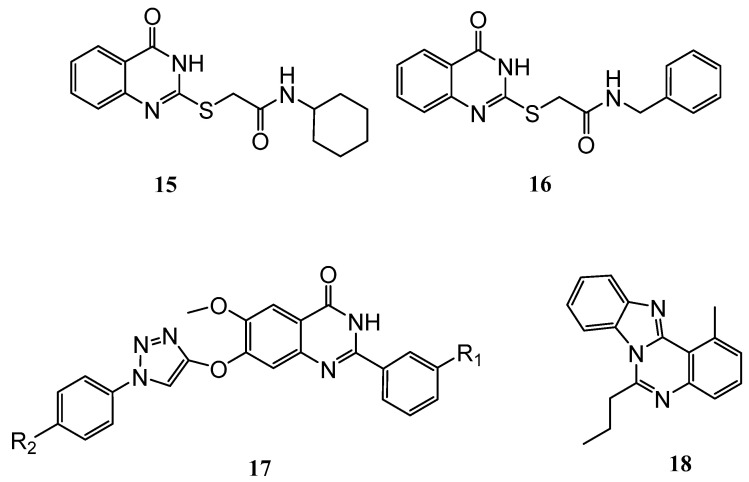



Thirty-two compounds were synthesized using benzimidazo quinazoline as a backbone and tested for their efficacy as anti-tubercular compounds. The compound 2-Methyl-6 propyl benzo [4,5] imidazo [1,2-c] quinazoline (**18**), was found to be the most effective against M. tuberculosis with an MIC value of 0.78 µg/mL [19].

### 3.3. Anticancer Activity

A number of studies have investigated the anticancer activity of quinazoline and quinazolinone derivatives [20,21,22,23,24,25]. Different types of cancers and cell targets were examined to achieve a novel quinazolinone derivative possessing significant anticancer activity. Wani and coworkers investigated the anticancer activity of a quinazolinone-chalcone derivative by cell cycle arrest in pancreatic cancer cell lines [20]. Chalcone, and its derivatives, is a well-studied chemical moiety with various biological activities such as anti-inflammatory, antimalarial, and anticancer activities. Chalcones are devoid of the genotoxic effect of other anticancer drugs due to interactions with amino groups of nucleic acid. However, the combined chalcone moiety with quinazolinone led to an increase in anticancer activity and a decrease in genotoxic effects (**19**). Moreover, this study found that one compound significantly inhibited the growth of different cell lines including Ehrlich Ascites Carcinoma and Sarcoma-180. This research concluded that the anticancer activity of this derivative is exerted through the cell cycle arrest at the G2/M phase [20].

A further 2015 study investigated the synthesis and anticancer activity of fluorinated quinazolinone-sulphonamide hybrids (**20**). Eight novel quinazolinone-sulphonamide derivatives were synthesized and tested for their in vitro cytotoxic activity. All of the eight compounds showed significant activity against cancerous cells and had a safe profile on non-cancerous cells [21]. Additionally, a series of 2,3,6-trisubstituted-4(3*H*)-quinazolinones (**21**) were synthesized and tested in vitro for their anticancer activity against breast cancer, hepatocellular carcinoma, cervical cancer, and promyelocytic leukemia cell lines. All tested compounds showed anticancer activity [22]. The synthesis and anticancer activity of 2-thioxoquinazolin-4-ones (**22**) were investigated by Abuelizz et al. (2017). All synthetic compounds were tested and showed sufficient anticancer activity against different cell lines when compared to the gefitinib as a positive standard [23].

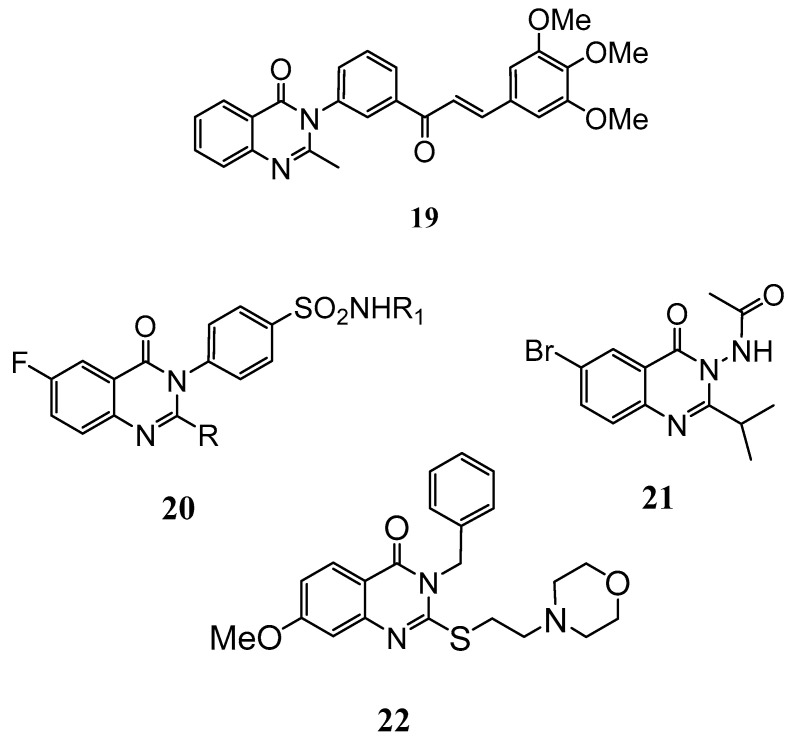



Another 2017 study investigated the synthesis, molecular docking, and anticancer activity of a 2-substituted mercapto-3-(3,4,5-trimethoxybenzyl)-4(3*H*)- quinazolinone analogue. This compound (**23**) was found to be the most potent of the tested derivatives. Additionally, a molecular docking study observed that this active compound binds to the epidermal growth factor receptor (EGFR) kinase enzyme [24]. Additionally, more recently, Nowar et al. (2018) examined a series of quinazolines and quinazolinones as apoptosis inducers. This study found that two quinazolinone compounds (**24** and **25**) are effective in inducing apoptosis in the HCT-116 cell line when compared to doxorubicin as a standard via interference with extrinsic and intrinsic apoptotic pathways. The authors also noticed that compound **25** showed a great selectivity to cancerous cells over compound **24** [25].

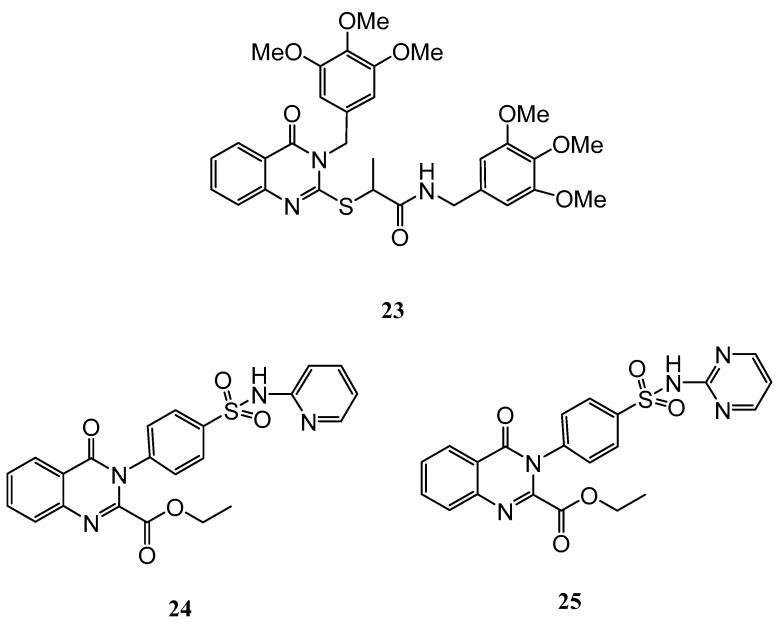



A series of novel quinazolinone derivatives were synthesized and examined for their cytotoxicity to the HepG2, MCF-7, and Caco-2 cancer cell lines in an MTT assay. One of these derivatives, compound **26**, was promising, with potent cytotoxicity against the Caco-2, HepG2, and MCF-7 cancer cells [26].

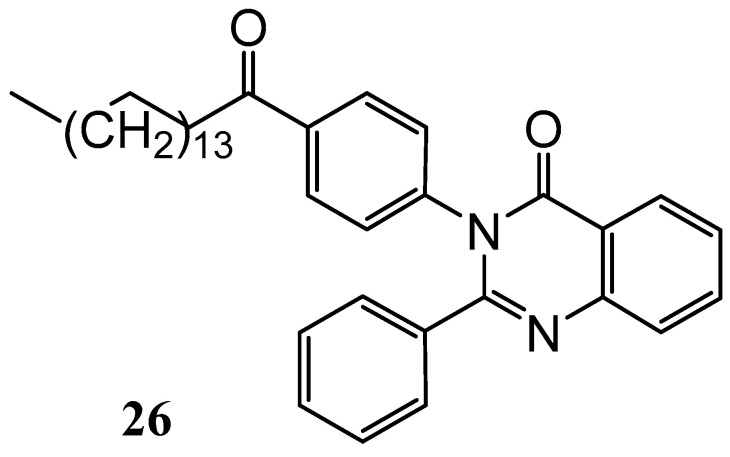



### 3.4. Activities on Central Nervous System (CNS)

#### 3.4.1. Anticonvulsant Activity

Quinazoline and quinazolinone derivatives have received great attention for their anticonvulsant activity throughout the years. The synthesis of a series of 2,3,8-trisubstituted-4(3*H*)-quinazoline derivatives has been discussed by Alazab and Altahir [27]. Their anticonvulsant activities against electrically induced seizures using maximal electroshock (MES) and chemically induced seizures using Pentylenetetrazole (PTZ) were investigated and compared with methaqualone and sodium valproate as a positive anticonvulsant standard. Compounds **27**, **28**, and **29** were found to have the most potent anticonvulsant activities with low neurotoxicity [27]. Moreover, a series of fluorinated quinazolinones were investigated in a 2014 study for their anticonvulsant activity against MES-induced seizures in a group of Swiss mice. Four out of eight compounds were found to be significantly active against MES-induced seizures. These four compounds have a halide at position 3 of the quinazolinone system similar to **30** [28].

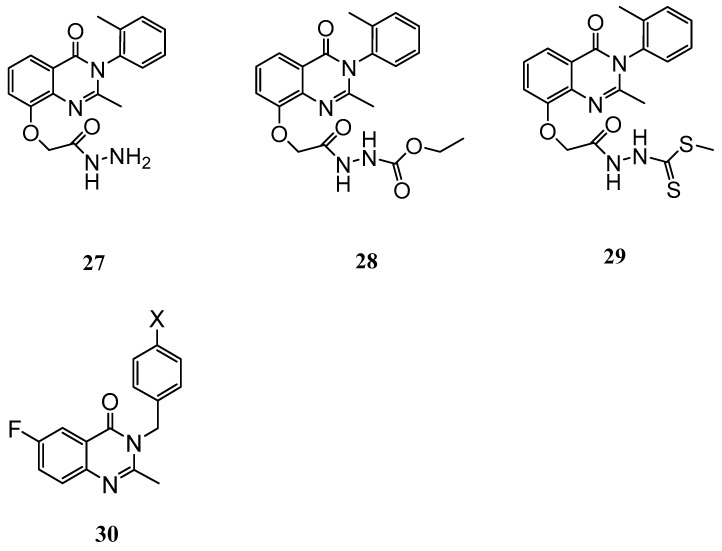



**Figure 3:**
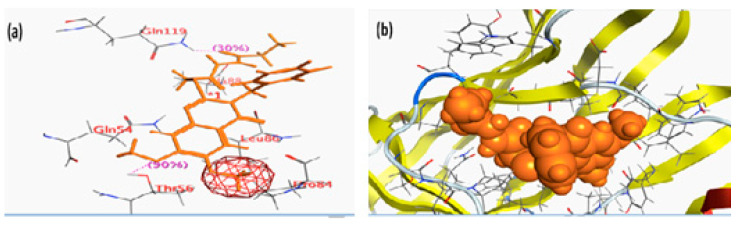
(**a**) The interaction and binding of the most active (orange) with amino acid residues in the diazepam-binding pocket of the GABAA receptor. (**b**) The aligned conformation of the most active compound (space-filled) occupying the diazepam-binding pocket of the GABAA receptor [29].

Additionally, a study by Alsalem et al. (2015) investigated the synthesis and anticonvulsant activity of a series of Hydrazine-carbothioamide, Benzenesulfonohydrazide, and Phenacylacetohydrazide analogues of 4(3*H*)-quinazolinone [29]. Four compounds were found to be effective with 100% protection against PTZ-induced convulsions. It was suggested in molecular docking studies as one of the most active compounds (**31**) as it displays agonistic behavior toward the GABA_A_ receptor [29], (Figure 3).

Patel et al. (2016) studied a series of quinazoline derivatives which were synthesized, virtually screened, and evaluated for anticonvulsant activity against electrically and chemically induced seizures. Compound (**32**) was found to possess the most potent anticonvulsant compound with relatively low neurotoxicity. SAR studies showed that the presence of a chlorine atom at position 7 on the quinazolinone system favors the anticonvulsant activity. 2-amino phenyl at position 3 increased the anticonvulsant activity [30]. Another 2016 study by Boshta et al. reported the synthesis and anticonvulsant activity of a novel series of 4(3*H*)-quinazolinone derivatives bearing oxadiazoles, thiadiazoles, and other moieties at position 3 (**33**). The activity of these compounds was compared to the phenobarbital activity. The study found six active compounds, all of which contained a 5-membered heterocyclic ring system in the substitution at position 3. Oxadizole is present in five of these active compounds, whereas the sixth compound contains a tetrazole ring in the substitution at position 3 [31].

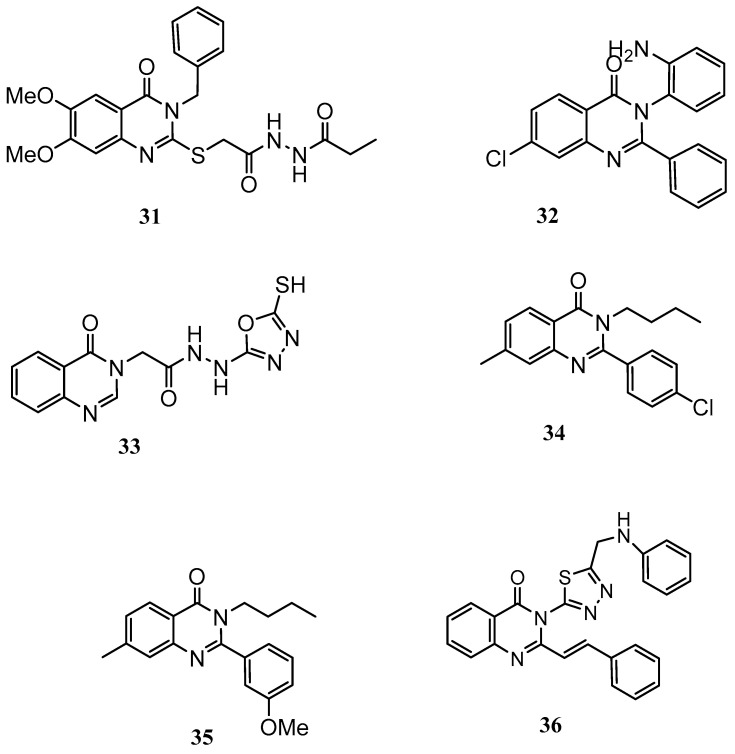



**Figure 4:**
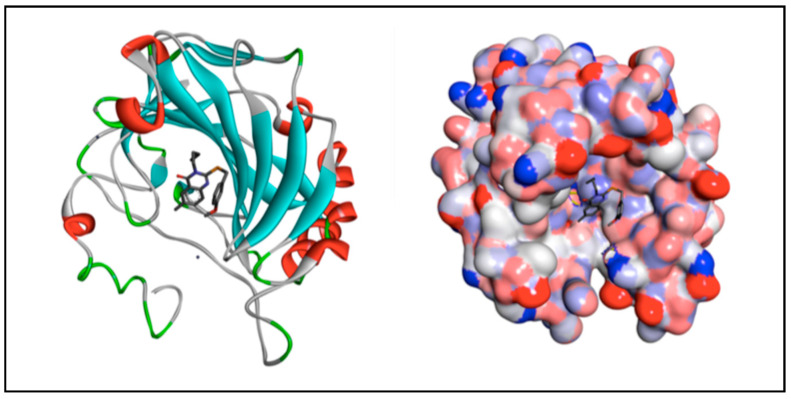
Docking of one of the active compounds in the active site of human carbon anhydrase II (left) and the electrostatic surface potential of human carbon anhydrase II in complex with the active compound [32].

Abuelizz et al. (2017) examined twenty-four quinazolinone derivatives for their anticonvulsant activity against chemically and electrically induced seizures. Two compounds proved to be the most active with 100% protection against chemically induced seizures (**34** and **35**). SAR studies followed by the molecular docking (Figure 4) of these two active compounds concluded the significant effect of butyl substitution at position 3 in preventing the spread of seizure discharge and raising the seizure threshold [32].

New derivatives of ((E)-3-(5-((sub-phenylamino) methyl)-1,3,4-thiadiazol-2-yl)-2styrylquinazolin-4(3*H*)-one are chemically prepared and evaluated for their anticonvulsant property. Eight synthesized chemicals have shown excellent anticonvulsant efficacy and protection against MES convulsion. These drugs can be expected to have better activity than phenytoin and carbamazepine but failed to show the effect after 4 h of administration [33].

#### 3.4.2. Anti-Alzheimer’s Activity

**Figure 5:**
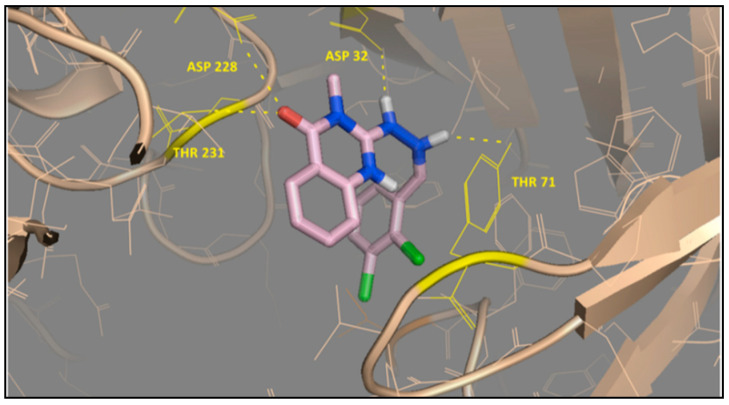
Docking of the most active compound 4 h in the active site of BACE1 [34].

Haghighijoo et al. (2017) investigated a β-site Amyloid precursor protein Cleaving Enzyme 1 (BACE1) and explained the importance of this protein as a significant target for the treatment of Alzheimer’s disease. Non-peptide inhibitors (quinazolinone-based hydrazones) of BACE1 were investigated using different enzymatic assays. Compound **37** was found to possess the highest activity to prevent the formation of β-amyloid which consequently protects the brain cells against apoptosis that help in delaying the appearance of Alzheimer’s disease. Molecular docking studies (Figure 5) confirmed a strong interaction between compound **37** and the key residues of the BACE1 active site [34].

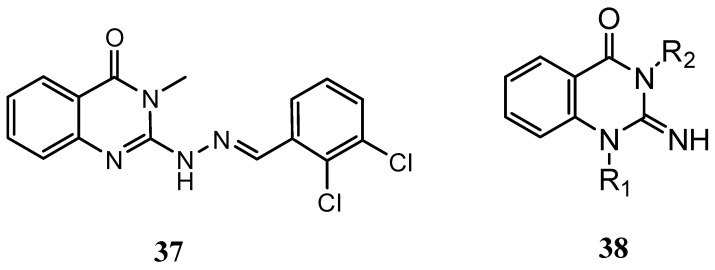



A series of novel quinazolinones were chemically prepared, docked, and predicted for their ADMET studies by Kachhadiya et al. In silico docking studies were performed to see the binding interactions of the synthesized compounds along with the approved drug Donepezil. The synthesized compound (**38**) was found to be introduced into the active site of AChE and orienting towards the active site, similar to Donepezil. All synthesized new quinazolinone derivatives present good BBB and CNS permeation, a low volume of distribution, and no negative effect on renal clearance [35].

#### 3.4.3. Antiparkinsonian Activity

Several 3-amantadinyl-2-[(2-substituted benzylidenehydrazinyl)methyl]-quinazolin-4(3*H*)-ones (5a–5l) were prepared and screened for their antiparkinsonian activity. Thiazolidinone derivatives showed more potent antiparkinsonian activity than azetidinone derivatives. Moreover, the substitution with the 3,4-dimethoxyphenyl group was found to be beneficial for antiparkinsonian activity. Albino rats were used in this study to investigate the effect of several 4(3*H*)-quinazolinone derivatives on tremors, rigidity, hypokinesia, and catatonia. Compounds that showed significant antiparkinsonian activity were evaluated further to assess their acute toxicity. The most active compound was 3-amantadinyl-6-bromo-2- [((3,4-dimethoxyphenyl)-4-oxo-thiazolidin-3-yl)methylamino]-quinazolin-4(3*H*)-one (**39**) [36].

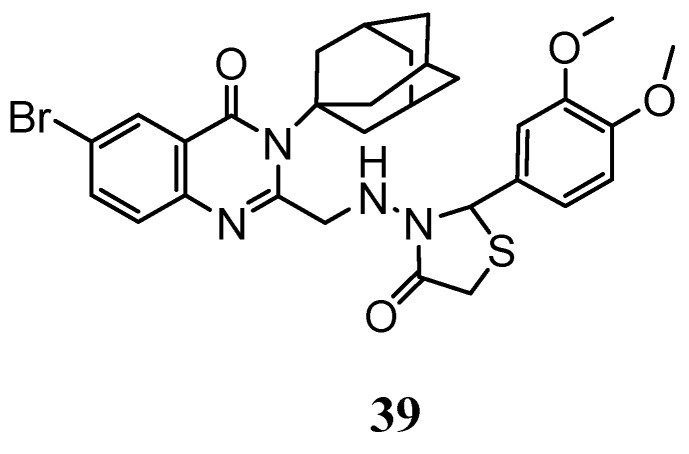



#### 3.4.4. Antidepressant Activity

Zhang et al. (2016) discussed the design, synthesis, and antidepressant activity of a series of novel 4-(substituted-phenyl) tetrazolo [1,5-*a*]quinazolin-5(4*H*)-ones and their derivatives with tetrazole and other heterocyclic substituents. Compound **40** was found to be very effective when compared to Fluoxetine [37].

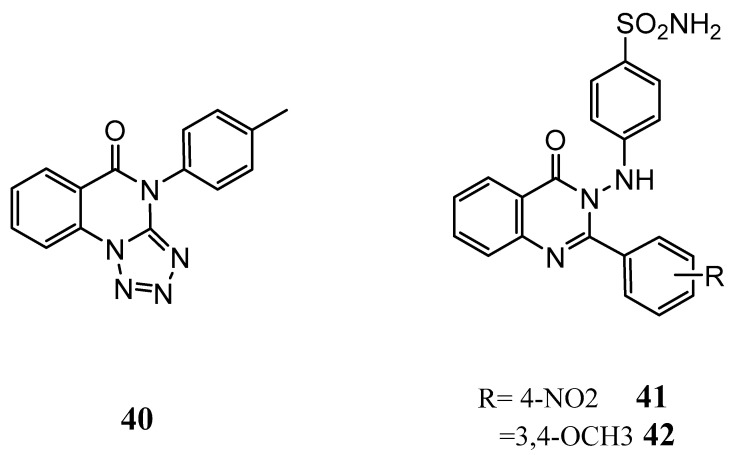



Interestingly, the researchers observed that compound **40** tends to increase the levels of noradrenaline and 5-hydroxytryptamine (5HT) while decreasing monoamine oxidase levels in mice brain tissue. This explains the underlying mechanisms of the abovementioned active derivative that might be involved in the regulation of brain monoamine neurotransmitter homeostasis [37]. In a study, 4-((2-(aryl)-4-oxoquinazolin-3(4*H*)-yl) amino) benzene sulfonamides were prepared and their MAOs inhibition potentials were tested through an in vitro fluorometric method. The most potent compounds **41** and **42** against MAO-A had IC50 values of 0.058 and 0.094 µM, respectively, whereas the standard moclobemide had an IC50 value of 6.061 µM [38].

### 3.5. Cardiovascular Activity

Eskandariyyan et al. (2013) synthesized and investigated a series of 2-(arylmethylthio)-3-phenylquinazolin-4-ones for their antiplatelet aggregation activity. Four derivatives were found to be significantly effective at preventing platelet aggregation against adenine diphosphate (ADP) and arachidonic acid-induced platelet aggregation in human plasma in vitro. Compound **43** displays the most effective derivative against platelet aggregation [39]. Moreover, a study by Singh et al. (2013) evaluated the cardiovascular activity of a series of 4(3*H*)-quinazolinones in vivo. Preliminary cardiovascular activity tests such as blood pressure and heart rate were carried out on albino rats. One of these derivatives, compound **44**, showed significant activity in lowering blood pressure and controlling heart rate [40]. A year later, Pathak et al. (2014) investigated the antihypertensive activity of eighteen 4(3*H*)-quinazolinone derivatives in vivo. Only seven derivatives noticed a significant hypotensive effect that led to the induction of bradycardia. Compound **45** is the most notable derivative that demonstrated this effect on hypertension [41].

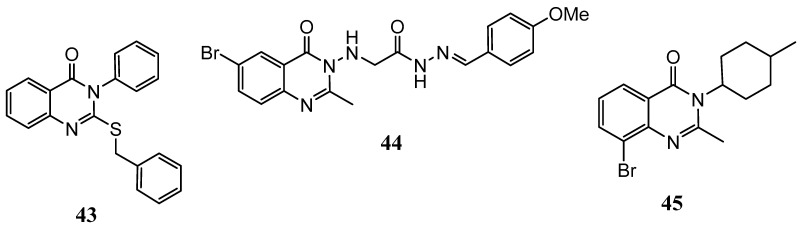



### 3.6. Antioxidants

Due to the excessive damage caused by the reactive oxygen (ROS) and nitrogen (RNS) species in humans, the need for new therapeutic agents, with superior efficacy to the known natural and synthetic antioxidants is crucial. Quinazolin-4-ones are known for their wide range of biological activities and phenolic compounds, possessing potent antioxidant effects. Therefore, connecting two active pharmacophores may lead to an increase in the antioxidant activity. Recently, Pele et al. (2022) synthesized four series of new hybrid molecules bearing the quinazolin-4-one and phenol scaffolds. Their antioxidant potential was evaluated in vitro. In fact, this study is considered as the basis for future research to evaluate the antioxidant activity of these compounds in experimental cell models and in vivo animal experimental models for these compounds that show good activity with a lack of toxicity [42].

## 4. Food and Drug Administration (FDA)-Approved 4(3*H*)-Quinazolinones

### 4.1. Quinethazone

**Figure 6:**
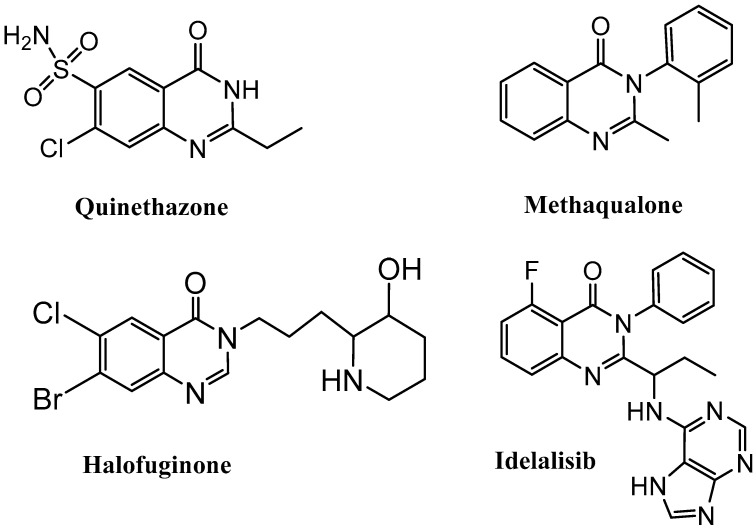
FDA approved 4(3*H*)-quinazolinones [43,44,45,46,47,48].

Quinethazone (Figure 6) is a thiazide diuretic prescribed for hypertension therapy. It was developed by Lederle in PutYear, marketed under the brand name Hydromox^®^, and approved by the FDA in 1963. This drug has great efficacy and a good safety profile with few side effects such as dizziness, dry mouth, and nausea [43,44].

### 4.2. Methaqualone

Methaqualone (Figure 6) is a sedative-hypnotic agent that is similar in effect to barbiturates, which were used for insomnia. In the 1980s, it was classified as a Schedule I drug by the FDA (i.e., a highly addictive substance with no current medical necessity in the US FDA). In fact, it is considered as a banned drug and is prohibited in the market. It has, however, been used illegally, especially in South Africa as a recreational drug as it acts as a central nervous system depressant [45].

### 4.3. Rhodoquinone

Halofuginone (Figure 6), or compound **10** above, is a febrifugine derivative that was developed in the 1960s. It was initially investigated for its antimalarial activity, in addition to other interesting biological effects that were reported in different animal studies. Halofuginone is an inhibitor of collagen type 1 synthesis. In in vivo studies, this compound showed a significant effect in the treatment of fibrosis. Furthermore, halofuginone inhibits tumor progression in some animal models such as bladder carcinoma. This compound is approved by the FDA for use in veterinary medicine for the treatment of protozoan parasites in cattle. Halofuginone was developed by Collgard Biopharmaceuticals and received FDA approval in 2000 as an orphan drug for the treatment of scleroderma. This unique compound is in clinical trials for different diseases. The oral administration of halofuginone has demonstrated a good safety and tolerability profile as well as promising responses in phase II of a study for the systemic treatment of recurrent superficial transitional cell carcinoma of the bladder. Halofuginone, in a slow-release formulation, is being evaluated in a phase II trial for Duchenne muscular dystrophy patients for safety, tolerability, and pharmacokinetics [11,46].

### 4.4. Idelalisib

Idelalisib (Figure 6) is a 5-fluoro-3-phenylquinazolin-4-one oral kinase inhibitor that was approved in 2014 for use in combination with rituximab for the treatment of relapsed or refractory chronic lymphocytic leukemia. It is also approved as monotherapy for relapsed follicular B cells and small lymphocytic lymphoma and is marketed under the brand name Zydelig^®^ by the pharmaceutical company Gilead Sciences. Idelalisib prevents the phosphoinositide-3 kinase signaling pathway and inhibits tumor cell proliferation, motility, and survival [45,47,48].

## 5. Investigational 4(3*H*)-Quinazolinones

### 5.1. Nolatrexed

**Figure 7:**
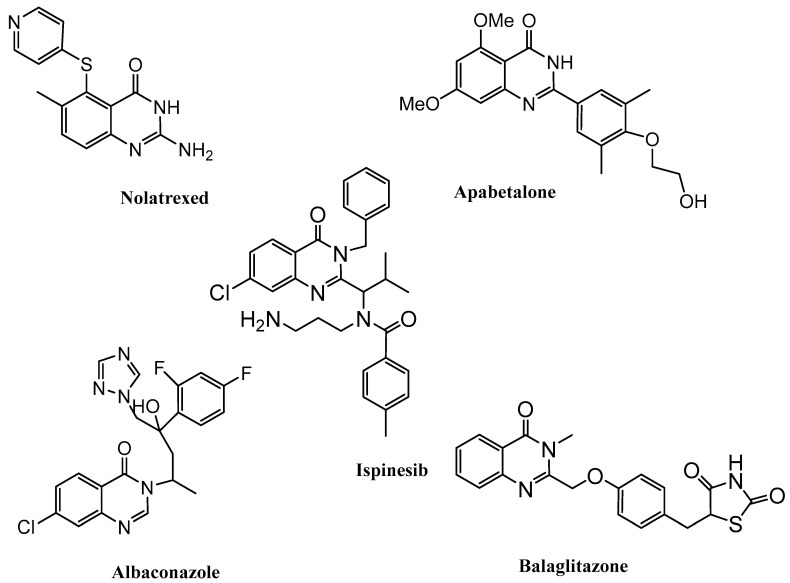
Investigational 4(3*H*)-quinazolinones.

Nolatrexed (Figure 7) is an investigational drug for the treatment of recurrent hepatocellular carcinoma. It is an antimetabolite agent that inhibits the enzyme dihydrofolate reductase, which converts dihydrofolate into tetrahydrofolate. This drug was developed by the Zarix company in 1999, which licensed worldwide rights of this drug to Agouron Pharmaceuticals. Agouron discontinued the development of Thymitaq based on the results obtained from analyses of phases II/III of trials. The findings concluded that this drug is not better than existing therapeutics to justify further development. This 4(3*H*)-quinazolinone derivative showed great efficacy in clinical trials with typical antimetabolite side effects demonstrating a short duration of action with low toxicity levels. Although this drug was granted an orphan drug status by the European Medicines Agency (EMA), the FDA refused to approve this drug in 2005 [45,49].

### 5.2. CapitalOne

Apabetalone (Figure 7) is an investigational drug developed by Resverlogix Corp. for the treatment of diabetes, atherosclerosis, and coronary artery disease. It is a selective bromodomain and extra-terminal domain inhibitor (BET). BET inhibition is an epigenetic mechanism that can regulate disease-causing genes. According to the developers, this drug is the world leader in a new class of drugs designed to regulate disease-associated proteins. Resverlogix started clinical trials of apabetalone in 2006, and it showed a significant reduction in major adverse cardiac events such as heart attacks and strokes in study participants. Recently, the company announced the start of a phase III clinical trial on this drug [45,49].

### 5.3. Ispinesib

Ispinesib (Figure 7) is being investigated for the treatment of lung cancer, breast cancer, renal cell carcinoma, ovarian cancer, liver cancer, melanoma, and head and neck cancer. It is developed by the Biopharmaceutical company, Cytokinetics. Clinical trials for different indications started in 2004, showing promising results in phase I/II clinical trials for some indications such as lung cancer, breast cancer, and liver cancer [45,49].

### 5.4. Albaconazole

Albaconazole (Figure 7) is an oral broad-spectrum antifungal agent under investigation for the treatment of onychomycosis (fungal infection of nails) and other fungal infections. This agent was discovered by Palau Pharma which licensed worldwide rights to Actavis in 2013. Clinical trials started in 2007 for different fungal infections. It was well tolerated in phase I/II clinical trials and resulted in high cure rates for onychomycosis. This antifungal drug is promising as it has an excellent safety profile and has demonstrated high efficacy [45,49].

### 5.5. Balaglitazone

Balaglitazone (Figure 7) was developed by Dr. Reddy’s Laboratories and Rheoscience for the treatment of type 2 diabetes mellitus. It acts as a peroxisome proliferator-activated receptor-gamma agonist. In 2010, phase III clinical trials for the treatment of type 2 diabetes mellitus were completed, and the results were very encouraging. However, research related to this agent has been discontinued [45,49].

## 6. Structure-Activity Relationship (SAR) of Quinazolinones

The purpose of including these studies in this review article is to gain a better understanding of the structure–activity relationships on the tested derivatives. Among hundreds of quinazolinone derivatives prepared every year around the world, the compounds included in this research will be too little to give an exact understanding of the complex behavior of quinazolinone rings with different targets in the body. Nonetheless, we try to shine a light on a way to better understand the main features of this group of compounds and how to manage their biological behavior by tiny modifications in their chemical structures. As such, the contribution of each position of the quinazolinone nucleus in various biological activities will be discussed:

### 6.1. Positions 2 and 3

The general pharmacophore of quinazolinones and each medical application should pass through positions 2, 3, or both. Indeed, most of the substitutions were found at positions 2 and 3 of the quinazolinone system; hence, these positions are likely to be more significant for various pharmacological activities. Antimalaria and anti-TB compounds are substituted at one position, either 2 or 3, as in compounds **9**–**17,** as well as the FDA-approved drug, Halofuginone. The other position is either substituted with a small group or completely not substituted.

Moreover, positions 2 and 3 are significant for anticancer derivatives; different heterocycles are used as substitutions at positions 2 and 3. In general, despite some exceptions, a successful cytotoxic drug could be obtained when a quinazolinone ring is substituted at position 2 with an alkyl side chain, and at position 3 with a bulky side chain such as a phenyl group (compounds, **19**–**26**).

**Figure 8:**
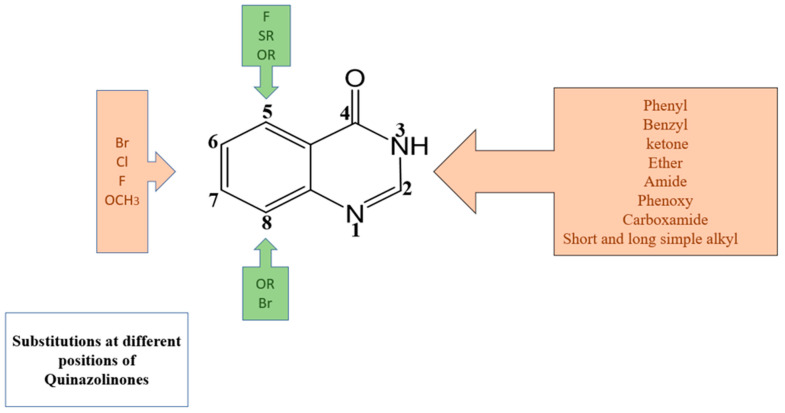
Substitutions at different positions of quinazolinones.

The groups substituted at positions 2 and 3 in addition to phenyl groups are mainly ketone, ether, amide, or carboxamide in the case of antimalaria and anti-TB compounds, **9**–**14**. However, in anticancer compounds, thioethers are mainly used as well as aryl ketones as seen with compounds **15**–**17** and the investigational drugs, Ispinesib and Naltrexed. Substituted phenyl or benzyl groups as well as short and long simple alkyl groups, thioethers, and carboxamides are mainly seen with anticonvulsant quinazolinones, whereas antidiabetic compounds contain a substituted phenoxy group at position 2, as seen in both investigational drugs Apabetalone and Balaglitazone (Figure 8).

### 6.2. Positions 6 and 7

These two positions are not necessary for activities as many quinazolinone derivatives are active in the absence of 6 and 7 substitutions (compounds **9**, **11**–**13**, **15**, **16**, **19**, **23**–**29**, **33**, **36**–**38**, and **40**–**43,** as well as the two FDA-approved drugs, Methaqualone and Idelalisib, in addition to the investigational drug, Balaglitazone). However, they play an important role in the pharmacokinetics of the compound, especially bioavailability and penetration to the CNS. On the other hand, substitutions at 6 or 7 modify the reactivity of the ring toward in vitro reactions; Halides, with their electron-withdrawing properties, increase the reactivity of the ring to combine with many substitutions on 2 or 3. The methoxy group, however, causes an opposite effect, reducing the reactivity of the ring toward many reactions. Therefore, in vivo, the addition or removal of such compounds could be a tool to manage the toxicity of quinazolinones (Figure 8).

### 6.3. Position 5

Position 5 is rarely substituted, it is specific to cytotoxic drugs, namely, protein kinase inhibitor Idelalisib and dihydrofolate reductase inhibitor Nolatrexed. It could be attached with an ether group in the structure of compound **11** that kills resistant strains of malaria, whereas substitution with a methoxy group gives Apabetalol, a cardiovascular effective drug.

### 6.4. Position 8

**Figure 9:**
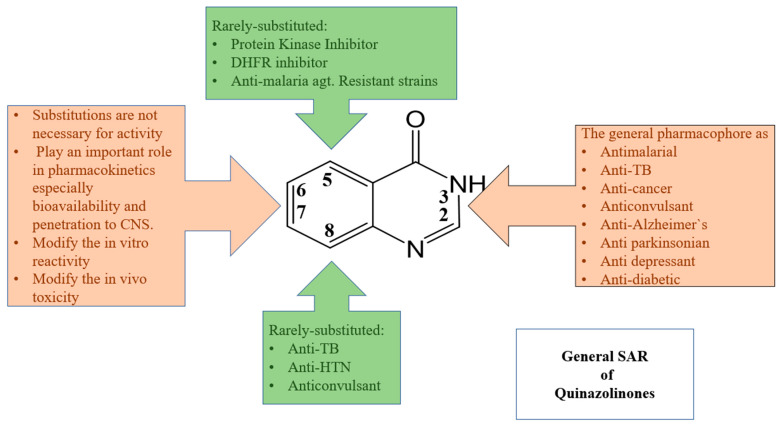
Summary of SAR of quinazolinones.

Position 8 is found in three compounds in this study, either brominated in the anti-TB compound 14, in the antihypertensive compound 45, or alkylated in three compounds with anticonvulsant activity (**27**–**29**). Figure 9 summarizes the SAR of quinazolinones.

## 7. Conclusions

Quinazolinones are considered a privileged structure in drug development for many reasons. The stability and relatively easy and straightforward synthetic methods of these compounds are significant reasons for scientists’ continuous interest in this moiety. The fact that they are lipophilic helps in the penetration of the blood–brain barrier, which makes them suitable for targeting the central nervous system for various illnesses.

Considering the reviewed studies, one can conclude that various modifications to the substitutions around the quinazolinone system alter the biological activity significantly due to the changes in its physicochemical properties. Furthermore, these metabolites might be useful as lead chemical compounds for the discovery of novel drugs and in the application of many syntheses. The discovery of novel natural phytomolecules from quinazolinone and the development of concrete applications still remain important and require organized and systematic studies.

## Data Availability

https://access.library.ksu.edu.sa/.

## References

[1] Kumar Tiwary B., Pradhan K., Kumar Nanda A., Chakraborty R. (2016). Implication of Quinazoline-4(3H)-Ones in Medicinal Chemistry: A Brief Review. J. Chem. Biol. Ther..

[2] Connolly D.J., Cusack D., O’Sullivan T.P., Guiry P.J. (2005). Synthesis of Quinazolinones and Quinazolines. Tetrahedron.

[3] Mahato A., Srivastava B., Nithya S. (2011). Chemistry Structure Activity Relationship and Biological Activity of Quinazoline-4 (3H)-One Derivatives. Inven. Rapid Med Chem.

[4] Horton D.A., Bourne G.T., Smythe M.L. (2003). The Combinatorial Synthesis of Bicyclic Privileged Structures or Privileged Substructures. Chem. Rev..

[5] Khan I., Ibrar A., Abbas N., Saeed A. (2014). Recent Advances in the Structural Library of Functionalized Quinazoline and Quinazolinone Scaffolds: Synthetic Approaches and Multifarious Applications. Eur. J. Med. Chem..

[6] Asif M. (2014). Chemical Characteristics, Synthetic Methods, and Biological Potential of Quinazoline and Quinazolinone Derivatives. Int. J. Med. Chem..

[7] Undheim K., Benneche T. (2009). Pyrimidines and Their Benzo Derivatives. Compr. Heterocycl. Chem. II.

[8] He L., Li H., Chen J., Wu X.F. (2014). Recent Advances in 4(3H)-Quinazolinone Syntheses. RSC Adv..

[9] Jiang S., Zeng Q., Gettayacamin M., Tungtaeng A., Wannaying S., Lim A., Hansukjariya P., Okunji C.O., Zhu S., Fang D. (2005). Antimalarial Activities and Therapeutic Properties of Febrifugine Analogs. Antimicrob. Agents Chemother..

[10] Zhu S., Wang J., Chandrashekar G., Smith E., Liu X., Zhang Y. (2010). Synthesis and Evaluation of 4-Quinazolinone Compounds as Potential Antimalarial Agents. Eur. J. Med. Chem..

[11] McLaughlin N.P., Evans P., Pines M. (2014). The Chemistry and Biology of Febrifugine and Halofuginone. Bioorganic Med. Chem..

[12] Laleu B., Akao Y., Ochida A., Duffy S., Lucantoni L., Shackleford D.M., Chen G., Katneni K., Chiu F.C.K., White K.L. (2021). Discovery and Structure-Activity Relationships of Quinazolinone-2-carboxamide Derivatives as Novel Orally Efficacious Antimalarials. J Med. Chem..

[13] Babu R.R., Naresh K., Ravi A., Reddy B.M., Babu V.H. (2014). Synthesis of Novel Isoniazid Incorporated Styryl Quinazolinones as Anti-Tubercular Agents against INH Sensitive and MDR M. Tuberculosis Strains. Med. Chem. Res..

[14] Lu W., Baig I.A., Sun H.J., Cui C.J., Guo R., Jung I.P., Wang D., Dong M., Yoon M.Y., Wang J.G. (2015). Synthesis, Crystal Structure and Biological Evaluation of Substituted Quinazolinone Benzoates as Novel Antituberculosis Agents Targeting Acetohydroxyacid Synthase. Eur. J. Med. Chem..

[15] Patel M.B., Kumar S.P., Valand N.N., Jasrai Y.T., Menon S.K. (2013). Synthesis and Biological Evaluation of Cationic Fullerene Quinazolinone Conjugates and Their Binding Mode with Modeled Mycobacterium Tuberculosis Hypoxanthine-Guanine Phosphoribosyltransferase Enzyme. J. Mol. Model..

[16] Mirchi A., Sizochenko N., Leszczynski J. (2018). Fullerene Quinazolinone Conjugates Targeting Mycobacterium Tuberculosis: A Combined Molecular Docking, QSAR, and ONIOM Approach. Struct. Chem..

[17] Murugesan D., Ray P.C., Bayliss T., Prosser G.A., Harrison J.R., Green K., Soares De Melo C., Feng T.S., Street L.J., Chibale K. (2018). 2-Mercapto-Quinazolinones as Inhibitors of Type II NADH Dehydrogenase and Mycobacterium Tuberculosis: Structure-Activity Relationships, Mechanism of Action and Absorption, Distribution, Metabolism, and Excretion Characterization. ACS Infect. Dis..

[18] Maddali N.K., Viswanath I.V.K., Murthy Y.L.N., Bera R., Takhi M., Rao N.S., Gudla V. (2019). Design, Synthesis and Molecular Docking Studies of Quinazolin-4-Ones Linked to 1,2,3-Triazol Hybrids as Mycobacterium Tuberculosis H37Rv Inhibitors besides Antimicrobial Activity. Med. Chem. Res..

[19] Jadhavar P.S., Patel K.I., Dhameliya T.M., Saha N., Vaja M.D., Krishna V.S., Sriram D., Chakraborti A.K. (2020). Benzimidazoquinazo-lines as new potent anti-TB chemotypes: Design, synthesis, and biological evaluation. Bioorg. Chem..

[20] (2015). nticancer Activity of a Novel Quinazolinone-Chalcone Derivative through Cell Cycle Arrest in Pancreatic Cancer Cell Line. J. Solid Tumors.

[21] Zayed M.F., Ahmed H.E.A., Ihmaid S., Omar A.S.M., Abdelrahim A.S. (2015). Synthesis and Screening of Some New Fluorinated Quinazolinone-Sulphonamide Hybrids as Anticancer Agents. J. Taibah Univ. Med. Sci..

[22] El-Hashash M., Morsy J., Azab M., Mahmoud N. (2016). Design, Synthesis and Anticancer Activity of Novel 2,3-and 2,4-Disubstituted Quinazoline and Quinazolinone Derivatives. Heterocycles.

[23] Abuelizz H.A., Marzouk M., Ghabbour H., Al-Salahi R. (2017). Synthesis and Anticancer Activity of New Quinazoline Derivatives. Saudi Pharm. J..

[24] El-Azab A.S., Abdel-Aziz A.A.M., Ghabbour H.A., Al-Gendy M.A. (2017). Synthesis, in Vitro Antitumour Activity, and Molecular Docking Study of Novel 2-Substituted Mercapto-3-(3,4,5-Trimethoxybenzyl)-4(3H)-Quinazolinone Analogues. J. Enzyme Inhib. Med. Chem..

[25] Nowar R.M., Osman E.E.A., Abou-seri S.M., El Moghazy S.M., Abou El Ella D.A. (2018). Design, Synthesis and Biological Evaluation of Some Novel Quinazolinone Derivatives as Potent Apoptotic Inducers. Future Med. Chem..

[26] Noser A.A., El-Naggar M., Donia T., Abdelmonsef A.H. (2020). Abdelmonsef, Synthesis, In Silico and In Vitro Assessment of New Quinazolinones as Anticancer Agents via Potential AKT Inhibition. Molecules.

[27] El-Azab A.S., Eltahir K.E.H. (2012). Synthesis and Anticonvulsant Evaluation of Some New 2,3,8-Trisubstituted- 4(3H)-Quinazoline Derivatives. Bioorganic Med. Chem. Lett..

[28] Zayed M.F. (2014). New Fluorinated Quinazolinone Derivatives as Anticonvulsant Agents. J. Taibah Univ. Med. Sci..

[29] Al-Salem H.S.A., Hegazy G.H., El-Taher K.E.H., El-Messery S.M., Al-Obaid A.M., El-Subbagh H.I. (2015). Synthesis, Anticonvulsant Activity and Molecular Modeling Study of Some New Hydrazinecarbothioamide, Benzenesulfonohydrazide, and Phenacylacetohydrazide Analogues of 4(3H)-Quinazolinone. Bioorganic Med. Chem. Lett..

[30] Patel H.M., Noolvi M.N., Shirkhedkar A.A., Kulkarni A.D., Pardeshi C.V., Surana S.J. (2016). Anti-Convulsant Potential of Quinazolinones. RSC Adv..

[31] Boshta N.M., El-Essawy F.A., Ammar R.M., Ismail A.E.H., Wahba N.E. (2016). Synthesis of Some New Quinazolin-4(3H)-One Derivatives and Evaluation of Their Anticonvulsant Activity. Mon. Chem..

[32] Abuelizz H.A., El Dib R., Marzouk M., Anouar E.H., Maklad Y.A., Attia H.N., Al-Salahi R. (2017). Molecular Docking and Anticonvulsant Activity of Newly Synthesized Quinazoline Derivatives. Molecules.

[33] Patel S. (2021). The Antiepileptic Effect of Synthesized Derivatives of Quinazoline-4(3H)-One. B R Nahata Smriti Sansthan Int. J. Phramaceutical Sci. Clin. Res..

[34] Haghighijoo Z., Firuzi O., Hemmateenejad B., Emami S., Edraki N., Miri R. (2017). Synthesis and Biological Evaluation of Quinazolinone-Based Hydrazones with Potential Use in Alzheimer’s Disease. Bioorg. Chem..

[35] Kachhadiya R., Prajapati D., Patel K., Prajapati N. (2022). Synthesis and Virtual Screening of Some Novel Quinazolinone Derivatives as Potent Cholinesterase Inhibitors against Alzheimer’s Disease. J. Drug Deliv. Ther. JDDT.

[36] Kumar S., Kaur H., Kumar A. (2012). Synthesis of New Azetidinonyl/Thiazolidinonyl Quinazolinone Derivatives as Antiparkinsonian Agents. Arab. J. Chem..

[37] Zhang H.J., Wang S.B., Quan Z.S. (2015). Synthesis and Antidepressant Activities of 4-(Substituted-Phenyl)Tetrazolo [1,5-a]Quinazolin-5(4H-Ones and Their Derivatives. Mol. Divers..

[38] Yamali C., Gul H.I., Sakarya M.T., Saglik B.N., Ece A., Demirel G., Nenni M., Levent S., Oner A.C. (2022). Quinazolinone-based benzenesulfonamides with low toxicity and high affinity as monoamine oxidase-A inhibitors: Synthesis, biological evaluation and induced-fit docking studies. Bioorganic Chem..

[39] Eskandariyan Z., Esfahani Zadeh M., Haj Mohammad Ebrahim Tehrani K., Mashayekhi V., Kobarfard F. (2014). Synthesis of Thioether Derivatives of Quinazoline-4-One-2-Thione and Evaluation of Their Antiplatelet Aggregation Activity. Arch. Pharm. Res..

[40] Singh N., Agarwal R.C., Singh C.P. (2013). Synthesis and Evaluation of Quinazolinone Derivatives for Cardiovascular Activities. Glob. J. Inc..

[41] Pathak S., Malhotra V., Nath R., Shanker K. (2014). Synthesis and Antihypertensive Activity of Novel Quinazolin-4(3H)-One Derivatives. Cent. Nerv. Syst. Agents Med. Chem..

[42] Sandler G. (1964). quinethazone, a new oral diuretic. Br. Med. J..

[43] Pele R., Marc G., Stana A., Ionuț I., Nastasă C., Tiperciuc B., Oniga I., Pîrnău A., Vlase L., Oniga O. (2022). Synthesis of New Phenolic Derivatives of Quinazolin-4(3H)-One as Potential Antioxidant Agents—*In Vitro* Evaluation and Quantum Studies. Molecules.

[44] Wishart D.S., Feunang Y.D., Guo A.C., Lo E.J., Marcu A., Grant J.R., Sajed T., Johnson D., Li C., Sayeeda Z. (2018). DrugBank 5.0: A Major Update to the DrugBank Database for 2018. Nucleic Acids Res..

[45] Pines M., Spector I. (2015). Halofuginone—The Multifaceted Molecule. Molecules.

[46] Wu P., Nielsen T.E., Clausen M.H. (2015). FDA-Approved Small-Molecule Kinase Inhibitors. Trends Pharmacol. Sci..

[47] PubChem Database National Center for Biotechnology Information. https://pubchem.ncbi.nlm.nih.gov.

[48] Niculescu-Duvaz I. (2001). Thymitaq (Zarix). Curr. Opin. Investig. Drugs.

[49] Hekal M.H., Abu El-Azm F.S.M. (2018). New potential antitumor quinazolinones derived from dynamic 2-undecyl benzoxazinone: Synthesis and cytotoxic evaluation. Synth. Commun..

